# Dr. Adam's Case of Small-pox

**Published:** 1805-10-01

**Authors:** Joseph Adams

**Affiliations:** Berner's Street


### To Dr. BRADLEY.

Sir,

THE following are the further particulars of the case of small-pox mentioned in the postscript of my former letter.

When the patient first arrived at the hospital, she had the small-pox full upon her. On being desired to show the cicatrix of vaccination, a scar was visible enough to be discovered across the ward. However, on a nearer view, it was easy to perceive that it wanted the true vaccine character. The cavity sloped from the edges, which were puckered; the bottom is every where smooth. On feeling it, a much greater induration was perceived than could be produced by the vaccine pustule, and which is evidently occasioned by the consolidation of substance in the parts below from the great injury they have sustained.

The following questions and answers will at once satisfy you that the new practice ought not to suffer by this event.

By whom were you inoculated?

By Mr. ———, at Wooburn, about six years ago, when the rest of the poor were inoculated gratis.

Did that gentleman see you afterwards?

No.

Was the appearance of your arm like that of the rest?

No. Their's healed without any trouble, but mine was so bad as to be for some time in a sling, and a large core came out; after which it healed, but very slowly.

From this account it is evident, that instead of the true effects from vaccination, violent inflammation and slough took place. In such a state of parts no benefit could have been expected from vaccination, which ought always to be repeated if the progress of the disease, or series of appearances, materially differ from their established laws. For though I am aware many people have been pronounced safe both after variolation and vaccination, in whom the symptoms have been unusually slight, yet to me it appears that this confidence has been founded on fallacious grounds. Some inoculators say they have exposed such patients to the severest tests of the casual and artificial disease, and they have resisted both. But this only proves that they were not susceptible *at that time*, which might have been fairly inferred from the peculiar appearance of the inoculated part. It does not however follow, that at a future period they may be in a similar state. I would therefore, in all such cases, advise a subsequent insertion at a more distant period, and when the patient appears in perfect health.

Another remark I would add is, that though we cannot but admire the laudable zeal of gratuitous inoculators, whether of the profession or others, yet in so serious an undertaking as to pronounce a person safe from a loathsome and dangerous disease the greatest attention is certainly necessary. I mean not to accuse the practitioner in the case I have just related. It cannot be doubted that it was the woman's fault her arm was not properly examined; for, besides that she was probably instructed for that purpose, the unusual appearance was of itself a sufficient reason for applying to a professional gentleman, and none could be so proper as the vaccinator. But we have met with too many other instances, where it has been thought sufficient to vaccinate and leave the rest to chance. Though the greater part of these have done well, yet this is not a sufficient excuse for such inattention. Whoever undertakes to vaccinate, should first be made master of all the few varieties in the appearances of the arm. Drawings and paintings are insufficient for this purpose, not only because the eye is so easily deceived by probable similarities, but because, uniform as the pustule is, there are a few varieties which have not yet been described. To such as wish for practical instructions, I shall be ready at any time to exhibit all the varieties which are daily occurring at the Hospital. These should be learned in all their stages, and the vaccinator should not fail to see his patient at least four times during the progress of the business; this even in the customary form of the disease: wherever anomalous symptoms occur, the examination should be proportionally more frequent. Though vaccination, compared with variolation, is so slight that it can scarcely be called a disease, yet this very circumstance renders the greater attention necessary, inasmuch as every peculiarity, excepting in the figure of the pustule, is less striking. The figure is certainly more uniform than in small-pox, but it is liable to some variety; it is also quicker in its progress, and more transient in some of its characteristic tic appearances; I would therefore not only advise every vaccinator to keep a register of his cases, but to distinguish particularly what he receives as the report of his patient or others, from what he sees himself. When we consider the magnitude of the object, no friend to the discovery can think much of these minute attentions; and when we consider the industry of its opponents, no one will say they are unnecessary. I remain, &c.

*Berner's Street, Sept.* 6, 1805. JOSEPH ADAMS.

